# A Badiaga Case

**Published:** 1887-11

**Authors:** J. A. Biegler

**Affiliations:** Rochester, N. Y.


### A BADIAGA CASE.

J. A. Biegler, M. D., Rochester, N. Y.

Miss E. D.; had suffered, not continuously, for several months, from a severe pain in RIGHT EYEBALL, which EXTENDED TO FOREHEAD over this eye, AND THEN TO TEMPLE. Always worse in the afternoon.

March 9th, 1887, prescribed Badiaga$^{2c}$, not having a higher potency, three powders to be taken every morning. On the 23d of the same month, after a severe cold, the pain returned and the prescription repeated, and has remained free from pain to this time. See *Allen's Encyclopædia.*—*Clinical Bureau, I. H. A.*

